# Round the Hospitals

**Published:** 1918-03-09

**Authors:** 


### ROUND THE HOSPITALS.

THE period of suspense in regard to the fate of the devoted nurses on board the *Glenart Castle* may be considered at an end. There is now no more hope that the boats which put off into the high seas running when the vessel was struck can have reached land. So far as the death-roll of the nurses is concerned, it is one of the biggest tragedies of the war. Use had probably blunted the minds of those on board to the danger they ran from treachery, and all were peacefully at rest when the

☛ Previous articles appeared December 8, 15, 22, January 5, 12, 26, February 2, and March

**ROUND THE HOSPITALS—(*continued*).**

blow came. It is stated that the matron, Miss Beaufoy, had travelled 60,000 miles by sea since the beginning of the war, and had had altogether over 30,000 patients under her care. The perfect discipline on board is demonstrated by the fact that within the seven minutes which elapsed before the vessel sank all, or nearly all, those on board were got into the boats. All honour to the deathless valour of those whose lives are sacrificed in the succour of the wounded! Were there any among these nurses who had relatives dependent on them? And, if so, can any grant or pension be obtained for them, as in the case of sailors or soldiers who are killed on duty? A question should be put to the Chancellor of the Exchequer.

---

The world has heard with dismay, not, alas! with surprise, of the deterioration in the character of German women evidenced in the nurses' barbarity towards English prisoners of war. There are writers who would fain persuade us that it is our duty to work ourselves up into a mood of concentrated fury at the repeated tales of German cold-blooded cruelty until, like these miserable Teutonic nurses, we become inaccessible to reason or duty. This is the doctrine of hatred which it is common to hear preached in the press and in the drawing-room. But always at the root of barbarity towards the helpless lies a seed of blind terror. The German women tormented and execrated English prisoners because they had been taught to fear the English. First fear, then hatred, then cruelty; that is the sequence of things. And, thank Heaven! it is not in British nurses to take one step on this road to moral ruin. To be fearless, calm, and strong in the teeth of unexampled persecution has been the lesson which English soldiers and English nurses have been set to demonstrate. Is not this "English way" the very antithesis of craven hatred?

---

General Sir William Robertson had some stirring words to say at Lincoln on March 2 when he opened the 4th Northern General Hospital, of which Miss Sheppard, R.R.C., is principal matron. His remarks about the devoted and sympathetic services of women in the war, services "which have never been surpassed and which make one proud to think that one belongs to the same race as British women," were warmly appreciated by every hearer. No doubt he is aware that there are no places in which *moral* is better maintained in the teeth of much overstrain than in our military hospitals, large and small. The spirit which pervades the entire *personnel*, professional and amateur, in these establishments is for the most part exactly that cheerful determination to keep going at any cost "for the duration" which the General told his audience in another part of the town was essential for the victory of the Allies.

---

We are glad to note the lively interest evoked among members of the College of Nursing in the election of the new Council next month. The privilege of nominating members of the Council, now placed within the reach of every member, is a very important feature in the scheme, which secures freedom of choice. Those who wish to nominate candidates for election must observe the precautions, first, of choosing only as candidates nurses who are actually members of the College; next of obtaining the consent in writing of any nurse they may nominate to accept office should she be elected; thirdly, of securing the concurrence of another member of the College to second their candidate. All the twelve vacancies are to be filled by open nomination, eight representing England and Wales, two representing Scotland, and two Ireland.

---

When the nomination-papers have been received, ballot-papers will be sent out containing the names of the candidates suggested for office by the members. By this means every member may record her vote through the post. Thus the election is to be carried out in the only satisfactory manner by the whole body of members, including most of those serving abroad it is hoped to reach, although, of course, some are too far off to receive notice in time. Undoubtedly all this entails a great deal of labour at the central offices, but the task is lightened, we understand, by a band of voluntary helpers—past and present matrons, sisters, and nurses gladly giving an hour or two (some of them many days) to the work of folding and addressing the 16,000 papers and envelopes.

---

The Manchester Centre of the College of Nursing has made a good start, and the appointment of Miss Smith, R.R.C., as local representative is thoroughly popular. At Liverpool Miss Constance Worsley, matron of the Infirmary for Children, has been appointed Acting Honorary Secretary to the Centre. There is a friendly rivalry between the two first Centres, and the scope for their activities in both localities is sufficiently wide. It is not given to every group of nurses who unite for professional purposes to make history, but that is what Liverpool and Manchester nurses are doing. They are giving a lead which the rest of the country will follow in due course.

---

The forty-third annual report of the Trained Nurses' Annuity Fund is, like its predecessors, a very disappointing affair. Thirty-five annuities were granted during 1917, on which a total sum of £607 was spent. This works out at an average of £17 7s. to each annuitant, and we are bound to confess that this system of administration appears to us to be toying with the necessities of disabled women. Would it not be wiser to pay half the number of annuities and double the amounts? This year the number of annuitants is to be raised to forty. The nursing staffs of the naval and military hospitals have contributed £561 towards endowing a Queen Alexandra (War Memorial) Annuity, and the Territorial Force nursing staffs have given

**ROUND THE HOSPITALS—(*continued*).**

£592 towards a King Edward VII. Memorial Annuity. It is abundantly clear that members of the nursing profession have not the means to provide adequately for their disabled sisters, of whom the report already quoted declares there are so many already that "a hundred annuities will not cover all pressing cases."

THE report of the matron at the Royal Infirmary, Bradford, submitted at the annual meeting of the subscribers is typical of hospital difficulties and triumphs throughout the country. Of the seventeen nurses who completed their training and went out from the hospital last year nine are serving in military hospitals. The greatest difficulty is experienced in keeping the nursing staff up to sufficient strength for the needs of the establishment, while emergency leave granted to any of its members entails much anxiety and re-arrangement. The matron pays a deserved tribute to the courage and devotion of her staff, who have carried on well and efficiently, in spite of all drawbacks. It is not surprising that the number of candidates shows an alarming decrease, for the competition for promising young women is intense and increasing. But a fall from 237 in 1916 to 158 in 1917 is certainly alarming. We are not afraid that the nursing profession under present conditions may not hold its own, but it is high time steps were taken to open up a more stable prospect for those who enter it.

AT a meeting of the London Insurance Committee held on February 28 reference was made to the difficulty experienced by the Metropolitan Asylums Board in obtaining nurses. A speaker attributed this difficulty to the long and arduous training the nurses had to go through. "The work was very hard and the pay contemptible." There are other causes, and one is the uncertain future prospect of these nurses should they not desire to remain in the service of the institutions under the Board. But, pending a thorough overhauling of the nursing system, why not start by raising salaries, as hospitals are doing in every direction?

THE matron of the Salisbury General Infirmary informs us that her committee had the matter of revising salaries for the nursing staff before them early last year. The probationers now receive £10, £15, and £20 in their first, second, and third years respectively, with an additional £5 on the completion of their training. Ward sisters receive £45; the home sister, night sister, and theatre sister £50. After fifteen years' service a pension is granted.

ADMIRERS of William Blake ought on no account to miss the chance of seeing the large collection of his hitherto almost unknown drawings belonging to the Linnell family, which is to be sold at Christie's on March 15. The drawings will be on view at the auction rooms on Tuesday, Wednesday, and Thursday in that week. They include the designs for Dante's "Inferno," only seven of which have been engraved, and a number of other highly interesting works in pen, pencil, and colour. It is earnestly to be hoped that this magnificent collection may be retained in this country and become accessible to the public after the obscurity which has so long hidden it from view.

"WANTED, matron and friend to carry on small country cottage hospital (seven beds); no servant kept; occasional help by charwoman." So runs an advertisement in the personal column of a daily paper. However the new matron arranges her scheme of work, it seems inevitable that some portion of the housework must fall to her lot, for the friend, with the doubtful help of an occasional "char," can hardly cook, clean, dust, and shop for nine people. But, should Matron have to give a hand with the cooking, what happens to the nursing? Even if a matron can be classed as a super-nurse, seven sick people would make a fair day's work. Then, presumably, Matron and the friend will need to take the air from time to time. On such an occasion, does the friend do the nursing and the matron do the housework, or does the charwoman do a little Sarah-Gamping in the old bad way? Much better would it be for the advertiser to persuade local folk to do their duty and provide funds for an adequate staff or to close the small country hospital and refer applicants to another institution in a neighbouring district where the fitness of things is better appreciated.

WE learn that thirty Nurses' Aides are working under the American Red Cross as assistants to the nurses who are doing special public health work in France. This is only one of the new developments which the war has brought about. In Massachusetts 100 women nurses have been granted commissions as second-lieutenants in the State Guard, and the Red Cross nurses already enrolled are protesting that their pay is inferior to that of the nurse lieutenants, or lieutenant nurses, although the latter are not exposed to war risks. The American Nursing Service is indubitably all alive, and its latest experiments are watched with much interest on this side of the water.

THE Cape Medical Council have adopted a minimum standard of general education for probationers desirous to enter hospitals for training. In the future no candidates for nurse training are to be accepted who have not reached Standard VII. It is not a very exacting test, but the important thing is to have some standard fixed below which probationers may not try the patience of sisters and vex the souls of the lecturers. That the question of fixing some standard of general education for British nurses lies ahead of the College of Nursing cannot be doubted. But neither here nor in South

**ROUND THE HOSPITALS—*(continued)*.**

Africa can the matter be settled once and for all time. The new Education Bill opens up such a vista of improvement in the mental equipment of all classes that our great-grandchildren may quite possibly look back to these illiterate days with some amusement.

The annual meeting of the Central Council for District Nursing in London, held on the 26th ulto., had little of interest on its programme. We are of opinion that this meeting each year ought to be full of interest, and that from it as a centre should radiate quickening forces which might be made of supreme value to district nursing throughout the metropolis. We shall be glad to hear that this point has been taken up by an active member of the Council with a view to energetic action.

In many institution gardens where great efforts were made last year to increase the potato-crop huge disappointment was caused by the appearance of potato disease. This tuber is, unfortunately, very liable to go wrong if the weather happens to be adverse in spring, and up to the present time diseased potatoes, though good enough for poultry and pigs, have spelled loss to the grower. Lately, however, a simple method has been discovered through which bad potatoes may be made to produce excellent starch and still leave a good residue over for his majesty the pig. Take about twenty raw potatoes, unfit for food, wash and pare them and put them to soak in a pan, just covered with cold water. Grate each potato on a coarse grater till all is pulp, and leave it in the water for an hour or so. Strain through a coarse cloth into another pan. A thick sediment of pure starch will be found to have settled at the bottom of the pan when the water has been poured away, and the residue or husk in the cloth will make good feed. Convalescent patients and children find interest in helping to prepare starch after this recipe. If the potatoes are not diseased but merely too small and poor for use in the ordinary way, they can be put through the same process, and the sediment will be potato-flour, which, when dried, can be used in bread-making, etc.

**☞ It is our invariable practice to pay for all items of news which we may publish. The minimum payment is 5s. Paragraphs of about twenty lines in length—that is to say, of 150 words or less—are preferred. Contributions should be addressed to The Editor, The Hospital, 28 and 29 Southampton Street, Strand, London, W.C. 2, and, to be in time for the current week's issue, should reach him by the first post on Mondays.**

